# Variability in the Qualitative and Quantitative Composition and Content of Phenolic Compounds in the Fruit of Introduced American Cranberry (*Vaccinium macrocarpon* Aiton)

**DOI:** 10.3390/plants9101379

**Published:** 2020-10-16

**Authors:** Ieva Gudžinskaitė, Elicija Stackevičienė, Mindaugas Liaudanskas, Kristina Zymonė, Vaidotas Žvikas, Jonas Viškelis, Rima Urbštaitė, Valdimaras Janulis

**Affiliations:** 1 Department of Pharmacognosy, Faculty of Pharmacy, Lithuanian University of Health Sciences, LT-50166 Kaunas, Lithuania; farmakog2@lsmuni.lt (I.G.); rima.urbstaite@lsmu.lt (R.U.); valdimaras.janulis@lsmuni.lt (V.J.); 2 Nature Research Center, Institute of Botany, LT-08406 Vilnius, Lithuania; elicija.stackeviciene@gamtc.lt; 3 Faculty of Pharmacy, Institute of Pharmaceutical Technologies, Lithuanian University of Health Sciences, LT-50166 Kaunas, Lithuania; kristina.gaivelyte@lsmuni.lt (K.Z.); Vaidotas.Zvikas@lsmuni.lt (V.Ž.); 4 Lithuanian Research Centre for Agriculture and Forestry, Institute of Horticulture, Babtai, LT-54333 Kaunas Distr., Lithuania; jonas.viskelis@lammc.lt

**Keywords:** American cranberry, antioxidants, phenolic compounds, biologically active compounds

## Abstract

The aim of this study was to determine the composition and content of phenolic compounds in ethanol extracts of eight different cultivars of American cranberry (*Vaccinium macrocarpon* Aiton) fruit using spectrophotometric and UPLC-ESI-MS/MS analysis and to evaluate the antioxidant activity in vitro of these extracts. The highest total amount of phenolic compounds evaluated via Folin–Ciocalteu spectrophotometry was detected in American cranberry fruit samples of the ‘Bain’ clone, and the highest total amount of flavonoids was found in samples of the ‘Drever’ and ‘Baiwfay’ cultivars. The highest total amount of the individual phenolic compounds (519.53 ± 25.12 mg/g DW) identified and quantitatively evaluated via chromatography was detected in samples of the ‘Searles’ cranberry cultivar. In the studied cranberry samples, the predominant phenolic compounds were hyperoside, quercetin, and procyanidin A2, while the amounts of other compounds were significantly lower. HCA and PCA revealed that ‘Woolman’, ‘Holliston’, ‘Pilgrim, and ‘Searles’ fruit samples had different quantitative content of phenolic compounds from other cranberry cultivars. Meanwhile, fruit of ‘Baiwfay’, ‘Drever’, ‘Bain’, and ‘Bergman’ were similar in their phytochemical profile.

## 1. Introduction

Over the last three decades, the use of botanical pharmaceutical preparations and dietary supplements has increased significantly and over 80% of the population worldwide relies on the effectiveness of botanical pharmaceuticals in primary healthcare [1]. According to the World Health Organization, traditional medicine is an important and frequently underestimated part of healthcare. A document passed in 2013 regulates the strategy of the use of traditional medicine for the period of 2014–2023. The aim of this strategy is to help the member states use the potential positive input of traditional medicine in disease prevention and treatment and to stimulate safe and effective use of traditional medicine [2]. 

Recently, plants that accumulate higher amounts of biologically active compounds have gained popularity. American cranberry (*Vaccinium macrocarpon* Aiton) is one such highly promising plant. This sort of cranberry naturally grows in the eastern and central regions of North America, but is increasingly commonly grown in Europe and other continents [3]. The fruit of American cranberry are especially valued for the variety of the biologically active compounds they contain. Research has shown that the fruit of American cranberry accumulate high amounts of phenolic compounds (anthocyanins, phenolic acids, flavan-3-ols, and flavonols), organic acids, and mineral substances [4,5,6]. Phenolic compounds in the fruit of American cranberry are especially important for human health, as they, acting as natural antioxidants, protect the body from diseases that develop because of damage caused by oxidative stress, including such diseases as cancer, cardiovascular diseases, or age-related degenerative disorders [7,8]. The oligomeric flavan-3-ol complex in American cranberry fruit is equally important, as it has an antibacterial effect and prevents the adhesion of pathogenic microorganisms to the lining of the urinary tract, thus protecting the body from various bacterial diseases of the urinary bladder and the urinary tract [9,10,11]. Research has also proven that American cranberry fruit prevent ulcer formation by inhibiting the strains of the ulcer-causing bacteria *Helicobacter pylori* [12].

The plant cultivar is one of the important factors that influence the synthesis and accumulation of biologically active substances in fruit. The aim of this study was to identify and compare the variability in the qualitative and quantitative composition and content of phenolic compounds in American cranberry (*Vaccinium macrocarpon* Aiton) fruit of different cultivars and the ‘Bain’ clone grown in Lithuanian climatic conditions, and to evaluate their antioxidant activity in vitro. The findings of the conducted evaluations will provide new knowledge and will be highly relevant in selecting the most promising American cranberry cultivars whose fruit accumulate the highest amounts of phenolic compounds—natural antioxidants, and the fruit extracts have an antiradical and reducing effect in vitro. These evaluations are especially important from the practical viewpoint, as they allow for selecting the most promising cranberry cultivars grown in Lithuania, helping to provide the customers with high-quality American cranberry fruit with a known composition or to use the fruit extract for the production of functional food, dietary supplements, or other innovative preparations with a specific biological effect.

## 2. Results and Discussion

### 2.1. Determination of Total Phenolic and Flavonoid Content

As the chemical composition of the edible fruits of different cultivars can vary considerably [13,14] it is very important to compare and assess the chemical composition of the American cranberry fruit of different cultivars and to ensure their quality. The diversity of the chemical composition of plants is an important characteristic that is used for the selection of garden and medicinal plants as well as for the evaluation of the quality of botanical raw material. The qualitative and quantitative composition and content of biologically active compounds in naturally growing and cultivated plants vary between different cultivars, different organs of the plant, or individual plants of the same cultivar, and thus studies of the chemical variability in biologically active compounds are especially important and relevant. 

**Figure 1:**
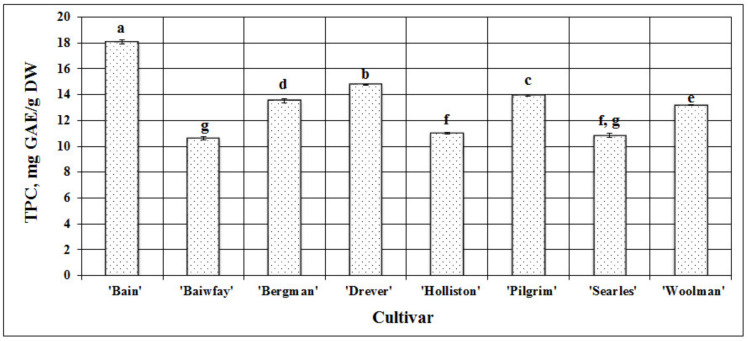
Variability in the total amount of phenolic compounds (TPC) in fruit samples of different American cranberry cultivars; different letters indicate statistically significant (*p* < 0.05) differences between the studied cranberry fruit samples.

In cranberry fruit, phenolic compounds are one of the predominant groups of biologically active compounds with a marked biological effect. The evaluation of the fruit samples of American cranberry cultivars grown in Lithuania showed that the total amount of phenolic compounds ranged from 10.61 ± 0.11 mg GAE/g DW (*p* < 0.05) in samples of the ‘Baiwfay’ cultivar to 18.06 ± 0.15 mg GAE/g DW (*p* < 0.05) in samples of the ‘Bain’ clone (Figure 1). Borowska et al. in their study found that the total amount of phenolic compounds in American cranberry fruit ranged from 192.1 mg/100 g in samples of the ‘Pilgrim’ cultivar to 374.2 mg/100 g in samples of the ‘Ben Lear’ cultivar (*p* < 0.05) [15]. Tikuma et al. in their study found that of the studied American cranberry cultivars, the highest amount of phenolic compounds (441 mg/100 g) was detected in the samples of the ‘Early Black’ cultivar [16]. In a study by Povilaitytė et al., the amounts of phenolic compounds in samples of American cranberry cultivars ranged from 192.3 mg/100 g to 676.4 mg/100 g [17]. 

We studied fruit samples of eight American cranberry cultivars and found the mean total amount of phenolic compounds for all samples to be 13.24 ± 0.1 mg GAE/g DW. To evaluate the variability in the quantitative content of phenolic compounds between fruit samples of different American cranberry cultivars, we calculated the coefficient of variation (CV), which was 18.89% and reflected the range of variation in the total amount of phenolic compounds. 

**Figure 2:**
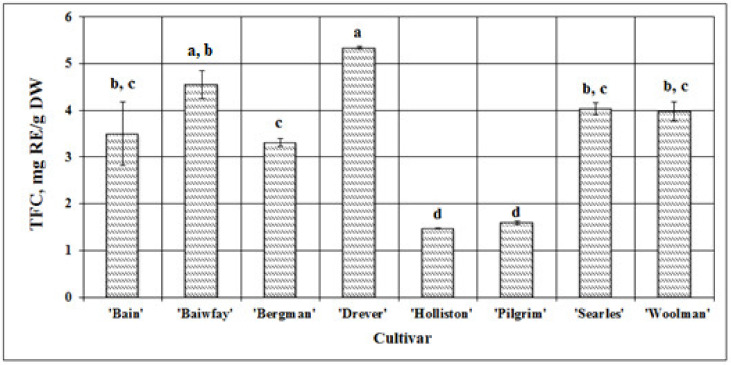
Variability in the total amount of flavonoids (TFC) in fruit samples of different American cranberry cultivars; different letters indicate statistically significant (*p* < 0.05) differences between the studied cranberry fruit samples.

Flavonoids are a large group of phenolic compounds with a pronounced effect. Determining the variability in the qualitative and quantitative composition and content of compounds of this group in the studied samples is an important step in the evaluation of the quality of botanical raw material. The total amount of flavonoids in cranberry fruit samples ranged from 1.47 ± 0.011 mg RE/g DW (*p* < 0.05) in samples of the ‘Holliston’ cultivar to 5.34 ± 0.026 mg RE/g DW (*p* < 0.05) in samples of the ‘Drever’ cultivar (Figure 2). In a study by Rudy et al., the total amount of flavonoids in American cranberry fruit samples ranged from 118 to 129 ± 3 mg QE /g DW [18].

We studied fruit samples of eight American cranberry cultivars and found the mean total amount of flavonoids for all samples to be 3.47 ± 0.18 mg RE/g DW. To evaluate the variability in the quantitative content of flavonoids between fruit samples of different American cranberry cultivars, we calculated the coefficient of variation (CV), which was 5.25% and reflected the range of variation in the total amount of flavonoids and a rather low variability in the total amount of flavonoids between fruit samples of different American cranberry cultivars. 

Data on the patterns of variation in the total content of phenolic compounds and flavonoids in American cranberry fruit are scarce. Therefore, this study provides new knowledge the total content of phenolic compounds and flavonoids in American cranberry fruit of the cultivars grown under Lithuanian climatic conditions, allows for the comparison of the obtained results with those of other studies, and is valuable for carrying out a search for promising, biologically active substance-accumulating botanical raw materials.

### 2.2. Identification and Quantification of Phenolic Compounds by UPLC-ESI-MS/MS

**Figure 3:**
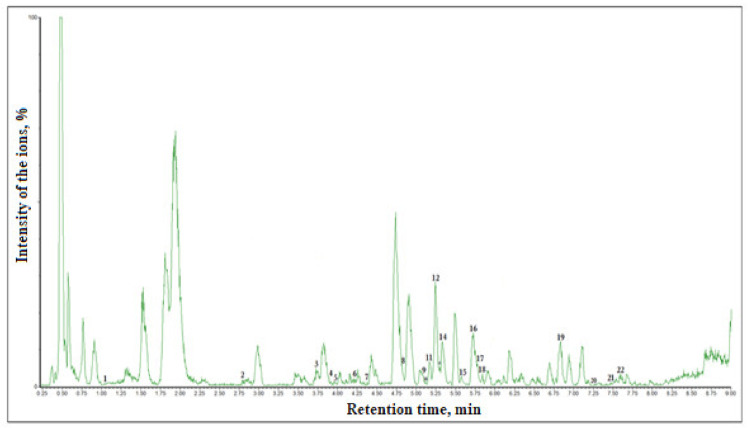
UPLC-ESI-MS/MS chromatogram of the ethanol extract of American cranberry (cultivar ‘Bergman’) fruit sample. The identified and quantitatively evaluated analytes are marked by numbers: 1—gallic acid, 2—neochlorogenic acid, 3—chlorogenic acid, 4—vanillic acid, 5—caffeic acid, 6—(–)-epicatechin, 7—procyanidin C1, 8—*p*-coumaric acid, 9—rutin, 10—luteolin-7-rutinoside, 11—ferulic acid, 12—hyperoside, 13—luteolin-7-O-glucoside, 14—procyanidin A2, 15—avicularin, 16—quercitrin, 17—isorhamnetin-3-O-glucoside, 18—phloridzin, 19—quercetin, 20—phloretin, 21—kaempferol, 22—isorhamnetin.

The application of the UPLC-ESI-MS/MS method and the use of the methodology developed by us in the analysis of phenolic compounds allowed for the identification of the qualitative and quantitative content of individual phenolic compounds and its variation in the fruit of different American cranberry cultivars. The following phenolic compounds of different groups were identified by applying this technique: monomeric and oligomeric flavan-3-ols, flavonols, dihydrochalcones, and phenolic acids (hydroxycinnamic and hydroxybenzencarboxylic acids). The quantitative content of the identified individual phenolic compounds is presented in Table 1, and the qualitative content of phenolic compounds is presented in a chromatogram in Figure 3. 

Flavonols are a group of flavonoids that is commonly found in the plant kingdom, and compounds in this group have a marked biological effect [19]. For this reason, it is important to determine the variability in the qualitative and quantitative composition and content of flavonols in botanical raw material. The following flavonols were identified: quercetin, isorhamnetin and kaempferol aglycones and their glycosides, and luteolin-7-O-glucoside. The highest total amount of the compounds of the flavonol group (370.38 ± 12.07 mg/g) was detected in fruit samples the ‘Searles’ cultivar of American cranberry, and the lowest (149.59 ± 5.62 mg/g)—in fruit samples of the ‘Pilgrim’ cultivar. Among the identified compounds of the flavonol group, quercetin glycosides—i.e., quercetin and its four glycosides (avicularin, quercitrin, hyperoside, and rutin)—predominated (Table 1). Research has shown that quercetin glycosides accumulated in American cranberry fruit have strong antioxidant [20,21], anti-cancer [22,23], and cardiovascular system-improving effects [24,25]. The anti-inflammatory effect of cranberry fruit, manifesting itself via the reduction of cytokine synthesis in macrophages and COX-2 expression as well as via the inhibition of TNF-α-dependent NF-*κ*B, is associated with the high amount of quercetin accumulated in the fruit [26].

In fruit sample extracts of all the studied American cranberry cultivars (except for ‘Searles’), hyperoside was the predominant compound, while in fruit samples of the ‘Searles’ cultivar, quercetin predominated, and the amounts of hyperoside were lower. The calculated coefficients of variation (CV) indicated the range of variability of each individual compound and showed that of all the compounds of the flavonol group, rutin (CV = 144%) had the greatest variability in fruit sample extracts of the studied American cranberry cultivars, while the variability of hyperoside was the lowest (CV = 22.6%). 

Extracts of American cranberry fruit samples were found to contain monomeric and oligomeric flavan-3-ols. Scientific literature indicates that in cranberries, flavan-3-ols (proanthocyanidins) of various degrees of polymerization have a strong antioxidant [27] effect and help prevent infections of the urinary bladder and the urinary system [20]. Flavan-3-ols in cranberry fruit inhibit the adhesion of the strains of *Escherichia coli* bacteria to the epithelial cells of the urinary tract [28] and suppress the effect of *Helicobacter pylori* bacteria on the cells of gastric mucosa [29]. Due to the aforementioned effects, cranberry fruit preparations are useful for the prevention and treatment of diseases caused by those pathogens [12,30]. Česonienė et al. indicted that oligomeric flavan-3-ols determine the organoleptic properties of cranberry fruit as well as their anti-inflammatory, antibacterial, and antiviral effects [31]. Proanthocyanidins in American cranberry fruit prevent periodontitis, and cranberry fruit preparations may also be used for the prevention of other oral diseases. Studies have shown that such preparations inhibit the growth of cariogenic bacteria [32], inhibit osteoclast differentiation and activity [33], and inactivate proteolytic bacterial enzymes [34]. In our studied extracts of American cranberry fruit samples, procyanidin A2 predominated, while the amounts of other compounds of this group were significantly lower. Procyanidin A2 is one of the main individual biologically active compounds in American cranberry [35]. In our studied samples, by the quantitative content, compounds of the flavan-3-ol group may be arranged in the following order, which was typical of fruit sample extracts of all the studied American cranberry cultivars: procyanidin C1 < (+)-catechin < (-)-epicatechin < procyanidin A2. The highest total amount of the identified compounds of the flavan-3-ol group (131.78 ± 5.29 mg/g) was found in the cranberry samples of the ‘Searles’ cultivar. Of all the compounds of the flavan-3-ol group in fruit samples of the studied cranberry cultivars, (-)-epicatechin demonstrated the highest variability (CV = 36.3%), while the variability in the amount of procyanidin C1 was the lowest (CV = 24.4%). Abeywickrama et al. studied different cranberry clones and found higher amounts of (+)-catechin and (-)-epicatechin than those found in our study [36]. 

Two compounds of the dihydrochalcone group were identified in American cranberry fruit samples—phloretin and its glucoside phloridzin. Both are known for their broad-spectrum biological effects. Research has proven that phloretin inhibits the proliferation of cancer cells [37] and has strong antihyperlipidemic [38,39] and anti-inflammatory effects [40,41]. Phloridzin slows down the development of age-related osteoporosis [42] and improves cognitive functions, and thus could be potentially useful in the treatment of Alzheimer’s disease [43] One of the most important biological effects of phloridzin is antidiabetic activity via the stimulation of lipid metabolism and body mass reduction. Due to this effect, phloridzin is used in the prevention and treatment of type 2 diabetes mellitus [44,45,46]. 

The highest total amount of the compounds of the dihydrochalcone group (8.00 ± 0.32 mg/g) was detected in American cranberry samples of the ‘Searles’ cultivar, and the lowest amount (2.53 ± 0.09 mg/g)—in cranberry samples of the ‘Pilgrim’ cultivar. In the samples of all the studied cranberry cultivars, the detected amount of phloridzin was significantly (by 31.3–51.8 times) higher than that of phloretin. The amount of phloridzin detected in the studied extracts varied widely, the coefficient of variation being 32.3%, while the variability in the amount of phloretin was lower (CV = 24.4%).

Phenolic acids as secondary metabolites are found in the majority of higher plants. In American cranberry fruit samples, compounds of the phenolic acid group have also been identified and quantitatively evaluated. Research has proven that hydroxycinnamic and hydroxybenzencarboxylic acids in American cranberry fruit have an antimicrobial effect and play a role in the biofilm inhibition and reduction of surface hydrophobicity of *E. coli* [47]. D’dharan and Neelakantan have indicated that gallic acid detected in American cranberry fruit had an antimicrobial effect by increasing the permeability of pathogenic microorganism strains and destabilizing the bacterial membrane via the chelation of divalent cations [48].

The total amount of phenolic acids in cranberry fruit samples ranged from 3.48 ± 0.12 mg/g (cultivar ‘Pilgrim’) to 10.68 ± 0.17 (cultivar ‘Bergman’). Among the identified phenolic acids, the group of hydroxycinnamic acids was most numerous, while only two compounds of the group of hydroxybenzencarboxylic acids—vanillic and gallic acids—were detected. In samples of the majority American cranberry cultivars, chlorogenic acid predominated in the group of phenolic acids. An exception were samples of the ‘Woolman’ cultivar, where, differently from other samples, vanillic rather than chlorogenic acid predominated. Among compounds of the group of hydroxybenzencarboxylic acids, vanillic acid predominated in samples of the studied American cranberry cultivars (except for the ‘Holliston’ cultivar). Its amounts were by 1.73–4.87 times higher than those of gallic acid were. The amount of gallic acid detected in fruit extracts of the ‘Holliston’ cultivar was higher than that of vanillic acid, compared to the chemical composition of the fruit extracts of other cranberry cultivars. In the studied fruit samples, the greatest variability was observed in the amount of neochlorogenic acid (CV = 139%), while the variability in the amount of ferulic acid was the lowest (CV = 21.7%). Kalin et al. studied aqueous extracts of lyophilized American cranberry fruit and found higher amounts of phenolic acids than those observed in our study [49]. The amount of *p*-coumaric acid found by these researchers was 13 µg/g, the amount of caffeic acid—5 µg/g, and the amount of ferulic acid—1.8 µg/g. Abeywickrama et al. who studied different cranberry clones also reported higher amounts of chlorogenic acid than those found in our study [36]. The differences of the obtained results might have been due to different methods of extract production used, differences in cranberry genotypes and climatic conditions, different conditions of cranberry cultivation, different ripeness of the fruit, and other factors. 

The highest total amount of the identified and quantitatively evaluated compounds (519.53 ± 25.12 mg/g) was found in American cranberry samples of the ‘Searles’ cultivar, and the lowest (204.36 ± 9.87 mg/g)—in cranberry samples of the ‘Pilgrim’ cultivar. The calculation of the coefficients of variation of different phenolic compounds showed that in the studied American cranberry samples, the variability in the total amount of phenolic acids was the highest (CV = 33.7%), while the variability in the total amount of flavan-3-ols was the lowest (27.5%). 

**Figure 4:**
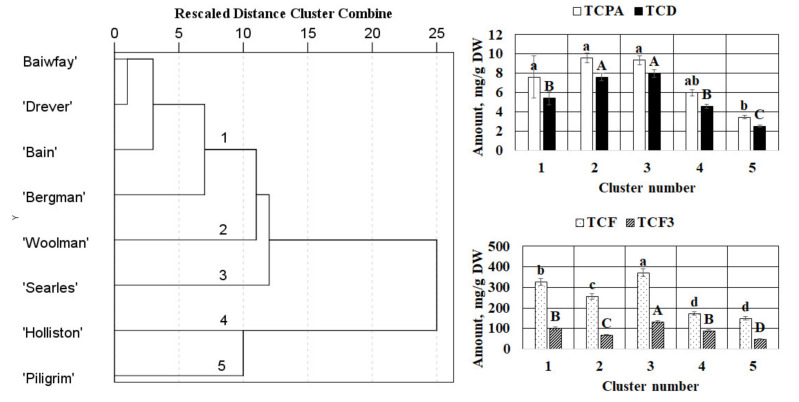
The dendrogram of hierarchical cluster analysis (HCA) of cranberry fruit based on the phytochemical composition and mean values of total contents of the identified compounds (mg/g DW) of clusters extracted using HCA. TCD—total content of dihydrochalcones; TCPA—total content of phenolic acids; TCF—total content of flavonols; TCF3—total content of flavan-3-ols. The different letters indicate significant differences between the values.

The hierarchical cluster analysis was applied for cranberry fruit based on the total contents of flavonols, flavan-3-ols, dihydrochalcones, and phenolic acids as clustering variables. As a result of the clustering of the fruit, the samples were grouped into five clusters (Figure 4). 

The first cluster grouped fruit samples of ‘Baiwfay’, ‘Drever’, ‘Bain’, and ‘Bergman’ cultivars. The samples forming the first cluster were characterized by the highest total content of phenolic acids. The fruit of the ‘Woolman’ cultivar formed the second cluster. The cluster was characterized by the highest total content of phenolic acids and dihydrochalcones. Fruit of the ‘Searles’ cultivar formed the third cluster. The corresponding fruit samples differed from the others by the highest total contents of the identified flavonols, flavan-3-ols, dihydrochalcones, and phenolic acids. Fruit of the ‘Holliston’ and the ‘Piligrim’ cultivars formed the fourth and the fifth clusters, respectively. Fruit samples of the ‘Holliston’ cultivar differed from the others by the lowest total content of flavonols while fruit samples of the ‘Piligrim’ cultivar were characterized by the lowest total content of flavonols, flavan-3-ols, dihydrochalcones, and phenolic acids (Figure 4). 

**Figure 5:**
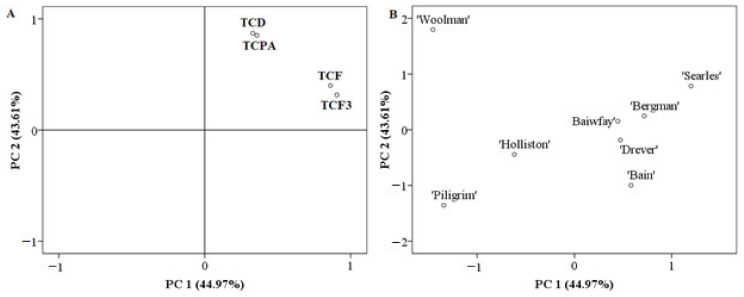
PCA loading (**A**) and score (**B**) plots of fruit samples of different cranberry cultivars. TCD—total content of dihydrochalcones; TCPA—total content of phenolic acids; TCF—total content of flavonols; TCF3—total content of flavan-3-ols.

A principal component analysis (PCA) was performed to detect similarities and differences between the analyzed samples according to the total content of flavonols, the total content of flavan-3-ols, the total content of dihydrochalcones, and the total content of phenolic acids. Figure 5 summarizes the PCA results based on the correlation matrix with PC1 and PC2, which explain 88.58% of the total variance in the data sets of cranberry fruit. The score plot models for fruit samples have shown relatively good separation between the cranberry cultivars (Figure 5).

In the PCA model, PC1 described 44.97% of the total variance of data and highly correlated with positive loadings of the total content of flavan-3-ols (0.906) and the total content of flavonols (0.861). PC2 accounted for 43.61% of the total variance and was characterized by the positive loadings of the total content of dihydrochalcones (0.871) and the total content of phenolic acids (0.851), chlorogenic acid (0.847), hyperoside (0.781), isoquercitrin (0.739), and rutin (0.685) (Figure 5B).

The fruit of the ‘Woolman’ cultivar was different from all the others. The clustering of this sample along the negative PC1 and positive PC2 can be explained by the high values of the total content of phenolic acids and dihydrochalcones and the low total content of flavonols and flavan-3-ols. Meanwhile, scattering of the fruit samples of the ‘Holliston’ and the ‘Pilgrim’ cultivars on the negative PC1 vs. PC2 space revealed the lowest total content of flavonols, flavan-3-ols, dihydrochalcones, and phenolic acids. Significant total contents of phenolic acids and dihydrochalcones scoring high in PC2 were found in fruit of the ‘Searles’ cultivar.

Moreover, fruit of the ‘Searles’ cultivar were located on the positive side of PC1 associated with the highest total content of flavonols and flavan-3-ols. Meanwhile, fruit samples of ‘Baiwfay’, ‘Drever’, ‘Bain’, and ‘Bergman’ cultivars demonstrated close positions in the PC1 vs. PC2 space indicating their similarity in the content of phenolic compounds. Fruit samples of these cultivars clustered closely near the zero-point indicating mean values of the total contents of phenolic acids and dihydrochalcones. On the other hand, fruit samples of ‘Baiwfay’, ‘Drever’, ‘Bain’, and ‘Bergman’ cultivars were located on the positive side of PC2 associated with the high total content of flavonols and flavan-3-ols.

### 2.3. Measurements of Antioxidant Activity in Extracts

After the evaluation of the qualitative and quantitative composition and content of phenolic acids and flavonoids in American cranberry fruit samples harvested from different cultivars grown under Lithuanian climatic conditions, it is important to examine and assess the antioxidant activity of their extracts in vitro. The results obtained during studies will be useful for the selection of promising American cranberry cultivars in order to provide consumers with products rich in antioxidants, will be useful for the assessment and standardization of the quality of botanical raw materials and their products, and will allow for predicting an antioxidant effect of American cranberry fruit sample extracts in vivo.

**Figure 6:**
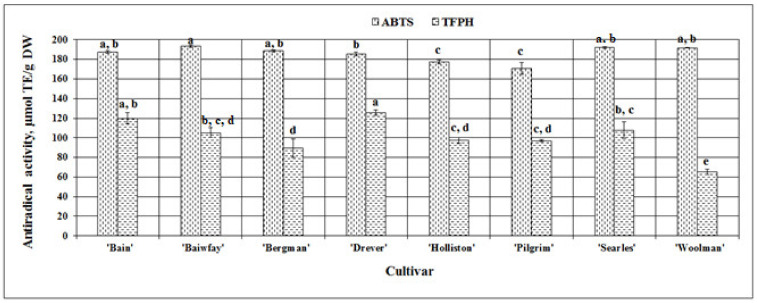
Variability in the antiradical activity in vitro in fruit sample extracts of different American cranberry cultivars; different letters indicate statistically significant (*p* < 0.05) differences between the studied cranberry fruit samples.

We evaluated the antiradical activity of the extracts of American cranberry fruit samples in vitro by using the ABTS^•+^ radical-cation scavenging assay. The strongest antiradical activity (193.63 ± 1.3 µmol TE/g DW) was observed in cranberry fruit extracts of the ‘Baiwfay’ cultivar, which did not differ statistically significantly from the antiradical activity of fruit extracts of the ‘Bain’ clone or ‘Bergman’, ‘Searles’, or ‘Woolman’ cultivars. The weakest antiradical activity evaluated by the ABTS assay was observed in American cranberry fruit extracts of the ‘Pilgrim’ and the ‘Holliston’ cultivars—respectively, 170.68 ± 5.95 µmol TE/g DW and 177.42 ± 2.19 µmol TE/g DW (Figure 6). The variability in the antiradical activity in vitro evaluated by this essay between cranberry fruit extracts was very low, with the calculated coefficient of variation being 4.29%. Floegel et al. in their study used the ABTS assay to evaluate antiradical activity of cranberry fruit in vitro and found that the mean antiradical activity was 119.6 ± 7.5 mgVCE/100g, and the coefficient of variation was 6.3% [50]. In a study by Abeywickrama et al., the antiradical activity in vitro of American cranberry fruit extracts of the ‘Pilgrim’ cultivar was 364.33 ± 1.39 µmol TE/g DW and was stronger than that observed in our study [36].

The evaluation using the TFPH assay showed that the strongest antiradical activity was observed in the cranberry fruit sample extracts of the ‘Drever’ cultivar and the ‘Bain’ clone (respectively, 125.66 ± 2.80 µmol TE/g DW and 119.91 ± 5.71 µmol TE/g DW), while the weakest antiradical activity was observed in the cranberry fruit sample extracts of the ‘Woolman’ cultivar (65.42 ± 2.73 µmol TE/g DW) (Figure 6). The coefficient of variation reflecting the variability in antiradical activity in vitro between cranberry fruit extracts of different cultivars was significantly higher (18.56%) than that calculated using the ABTS assay.

**Figure 7:**
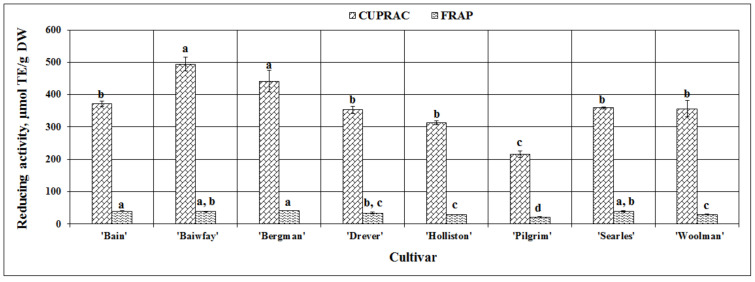
Variability in the reducing activity in vitro in fruit sample extracts of different American cranberry cultivars; different letters indicate statistically significant (*p* < 0.05) differences between the studied cranberry fruit samples.

When applying the FRAP assay, the strongest reducing activity in vitro was found in American cranberry sample extracts of the ‘Bergman’ cultivar (41.88 ± 0.18 µmol TE/g DW), yet it did not differ statistically significantly from that observed in fruit sample extracts of ‘Bain’, ‘Baiwfay’, or ‘Searles’ cultivars. The weakest reducing activity detected by using this assay (21.80 ± 0.37 µmol TE/g DW) was found in American cranberry sample extracts of the ‘Pilgrim’ cultivar (Figure 7). The coefficient of variation of the reducing activity of American cranberry fruit extracts evaluated using the FRAP assay was 19.78%. Çelik et al. in their study also evaluated the reducing activity of American cranberry fruit extracts using the FRAP assay and found that the mean reducing activity in American cranberry fruit samples was 12.61 mmol TE/kg FW [51]. In a study by Abeywickrama et al., the reducing activity in vitro of the extracts of American cranberry fruit samples of the ‘Pilgrim’ cultivar was by 1.12 ± 0.02 mmol TE/g DW stronger than that found in our study [36].

The strongest reducing activity evaluated via the CUPRAC assay was detected in the extracts of American cranberry fruit samples of the ‘Baiwfay’ and ‘Bergman’ cultivars (respectively, 493.87 ± 21.33 µmol TE/g DW and 441.71 ± 33.79 µmol TE/g DW), while the weakest reducing activity in vitro (215.15 ± 10.34 µmol TE/g DW) was found in cranberry sample extracts of the ‘Pilgrim’ cultivar (Figure 7). The calculated coefficient of variation was 22.79%

Namiesnik et al. applied the spectrophotometric CUPRAC assay when extracting American cranberry samples with different extractants and evaluated the reducing activity of the obtained extracts in vitro. They found the strongest reducing activity (49.38 ± 4.4 µM TE/g) in American cranberry extracts [52], but their results using the CUPRAC assay were significantly lower compared to those obtained in our study. Such differences might have been due to different extractants used, different cultivating conditions of American cranberries, different fruit harvesting years, different climatic conditions, and a number of other factors.

## 3. Materials and Methods

### 3.1. Plant Material 

In the evaluations, we used fruit samples of different American cranberry cultivars grown in Lithuanian climatic conditions in 2017: ‘Baiwfay’, ‘Holliston’, ‘Searles’, ‘Drever’, ‘Bergman’, ‘Woolman’, and ‘Pilgrim’, as well as a ‘Bain’ clone. American cranberry fruit samples were obtained from the institute of Botany of the Nature Research Center. The samples were lyophilized at the Institute of Horticulture, Lithuanian Research Center for Agriculture and Forestry. 

### 3.2. Chemicals

All the solvents, reagents, and standards used were of analytical grade and met all the set quality requirements. The following substances were used in the study: ethanol 96% (*v/v*) (AB “Stumbras”, Kaunas, Lithuania), the Folin–Ciocalteu reagent, sodium carbonate, gallic acid, acetic acid, ABTS (2,2’-azino-bis(3-ethylbenzothiazoline-6-sulfonic acid), Trolox (6-hydroxy-2,5,7,8-tetramethyl- chroman-2-carboxylic acid), potassium persulfate, copper (II) chloride, ammonia acetate, neocuproine, sodium acetate (Scharlau, Sentmenat, Spain), TPTZ (Carl Roth, Karlsruhe, Germany), iron (III) chloride hexahydrate (Vaseline-Fabrik Rhenania, Bonn, Germany), TFPH (trifluoperazine dihydrochloride), sulfuric acid, acetonitrile (Sigma-Aldrich, Steinheim, Germany), acetic acid (Lachner, Neratovice, Czech Republic); (+)-catechin, (-)-epicatechin, luteolin-7-o-glucoside, procyanidin C1, procyanidin A2, phloretin, kaempferol, rutin, hyperoside, quercitrin, phlorizin, avicularin, neochlorogenic acid, chlorogenic acid, isorhamnetin, ferulic acid, caffeic acid, gallic acid, vanillic acid, p-coumaric acid, hydrochloric acid, hexamethylentetramine, potassium chloride, aluminum chloride (Sigma-Aldrich), and isorhamnetin -3-O-glucoside (ExtraSynthese, Lyon, France).

### 3.3. Apparatures

Cranberry fruit were lyophilized in a lyophilizer Zirbus (Zirbus Technology GmbH, Bad Grund, Germany). Cranberry fruit samples were ground using a Retsch GM 200 electrical mill (Retsch GmbH, Haan, Germany). The raw material was weighed using a CP64–0CE electronic analytical scale (Sartorius AG, Göttingen, Germany). Extraction of phenolic compounds from cranberry fruit samples was carried out in a Sonorex Digital 10 P ultrasonic bath (Bandelin Electronic GmbH & Co. KG Darmstadt, Germany), and filtering was carried out by using a glass filter and a 2511 Dry Vacuum Pump/Compressor vacuum pump (Welch, Skokie, IL, USA). All the spectrophotometric measurements were carried out with a M550 UV/Vis spectrophotometer (Spectronic CamSpec, Garforth, UK). The analysis of phenolic acids, dihydrochalcone, flavan-3-ols, and flavonols in cranberry fruit was performed using an Acquity H-class ultra-performance liquid chromatography system (Waters, Milford, MA, USA) equipped with a Waters Xevo mass spectrometer.

### 3.4. Preparation of the Cranberry Fruit Samples

For the analysis, fruit of American cranberry grown in Lithuanian climatic conditions were used. The American cranberry fruit were frozen at −35 °C with air circulation. Following that, the fruit were lyophilized in a Zirbus lyophilizer (Zirbus Technology GmbH, Bad Grund, Germany) at 0.01 mbar pressure and –85 °C condenser temperature. The lyophilized fruit were then ground to powder. Their samples were stored in tightly closed vessels in a dark and dry place. The loss on drying of the raw material was determined by applying the technique described in the European Pharmacopoeia 07/2019:20232 [53].

### 3.5. Preparation of the Ethanol Extracts

During the study, 2.5 g (exact weight) of lyophilized American cranberry fruit powder was used, adding 30 mL of 2% HCl solution in 70% (*v/v*) ethanol and extracting in an ultrasonic bath for 40 min at 80 Hz frequency and 452 W power. The obtained extract was filtered, and the lyophilized American cranberry fruit mass remaining on the filter was then washed twice with 10 mL of 2% HCl solution in 70% (*v/v*) ethanol. The filtered extract was then poured into 50-mL measuring flasks, adding 2% HCl solution in 70% (*v/v*) ethanol up to the marking. Prior to the UESC analysis, the extracts were filtered through Carl Roth membrane filters (Carl Roth GmbH & Co. KG, Karlsruhe, Germany) with 0.22-µm pore size. 

### 3.6. Spectrophotometric Studies

#### 3.6.1. Determination of Total Phenolic and Flavonoid Content

The total phenolic content in the ethanol extracts of cranberry fruit was determined by using the Folin-Ciocalteu method [54], calculated from a gallic acid calibration curve, and expressed as mg/g gallic acid equivalent (GAE) per one gram of absolutely dry weight (DW) (mg GAE/g DW). The total amount of flavonoids in the ethanol extracts of cranberry fruit was determined using the described methodology [55], calculated from a rutin calibration curve, and expressed as mg/g rutin equivalent (RE) per one gram of absolutely dry weight (DW) (mg RE/g DW).

#### 3.6.2. Evaluation of Antioxidant Activity

Calculation of Antioxidant Activity of the Ethanol Extract of cranberry fruit. The antioxidant activity of the extracts was calculated from the Trolox calibration curve and was expressed as µmol of the Trolox equivalent (TE) per one gram of absolutely dry weight (DW). TE was calculated according to the following formula: $\mathrm{TE}\;=\;\frac{c\times V}{m}$ c: the concentration of Trolox established from the calibration curve (in µM); V: the volume of the extract (in L); m: the weight (exact) of the lyophilized fruit powder (in grams).

(1) *ABTS*^⋅+^*Assay*. During the evaluation, 3 mL of ABTS^⋅+^ solution was mixed with 10 µL of extracts. A decrease in absorbance was measured at λ = 734 nm [56]. A calibration curve (𝑦 = 0.00003𝑥−0.00360; R^2^ = 0.9714) was prepared using standard Trolox solutions of 8000 to 24,000 µmol/L concentration.
(2) *TFPH*^⋅+^*Assay*. 3 mL of TFPH^⋅+^ solution was mixed with 10 µL of extracts, and absorbance was measured at λ = 502 nm [57]. A calibration curve (𝑦 = 0.0000371𝑥 + 0.1471727; R^2^ = 0.9959) was prepared using standard Trolox solutions of 2000 to 16,000 µmol/L concentration.
(3) *CUPRAC Assay*. CUPRAC solution included copper (II) chloride (0.01 M in water), ammonium acetate buffer solution (0.001 M, pH = 7), and neocuproine (0.0075 M in ethanol) (ratio 1:1:1). During the evaluation, 3 mL of CUPRAC reagent was mixed with 10 µL of extracts. An increase in absorbance was recorded at λ = 450 nm [58]. A calibration curve (𝑦 = 0.0000222𝑥 − 0.0132677; R^2^ = 0.9995) was prepared using standard Trolox solutions of 2000 to 48,000 µmol/L concentration.
(4) *FRAP Assay*. FRAP solution included TPTZ (0.01 M dissolved in 0.04 M HCl), FeCl_3_ × 6H_2_O (0.02 M in water), and acetate buffer (0.3 M, pH 3.6) (ratio 1:1:10). During the evaluation, 3 mL of a freshly prepared FRAP reagent was mixed with 10 µL of extracts. An increase in absorbance was recorded at λ = 593 nm [59]. A calibration graph (*y* = 0.0000166𝑥 + 0.000950; R^2^ = 0.9926) was prepared using standard Trolox solutions of 400 to 24,000 µmol/L concentration.

### 3.7. Chromatographic Studies

The variability in the qualitative and quantitative composition and content of phenolic compounds in American cranberry fruit samples was evaluated using ultra performance liquid chromatography-mass spectrometry by applying the technique described and validated by Gonzalez–Burgos et al. (2018) [60]. Mass spectrometry parameters for the analysis of phenolic compounds are presented in Table 2.

### 3.8. Data Analysis

Data analysis was carried out using computer software Microsoft Excel 2016 (Microsoft, JAV) and SPSS Statistics 20 (IBM, JAV). During the analysis, we calculated arithmetic means and standard deviations of three repeated measurements. In order to evaluate the variability in the quantitative content between the samples, we calculated the coefficient of variation (CV). A univariate dispersion analysis model (ANOVA) was applied for determining whether the differences between the compared data were statistically significant. Differences between the samples were determined by applying Tukey’s multiple comparison test. Concerning the quantitative composition of the identified compounds, the tested samples were compared by applying hierarchical cluster analysis using squared Euclidean distances. Principal component analysis was performed taking into account factors with eigenvalues higher than 1.

## 4. Conclusions

In conclusion, the results of this study will provide new knowledge about the composition and content of phenolic compounds in fruit of American cranberries cultivated in Lithuanian climatic conditions and the antioxidant activity of their extracts in vitro, which will give a wide range of possibilities to employ these plants as a source of phenolic compounds. First, during pilot spectrophotometric evaluations, the highest total amount of phenolic compounds was found in American cranberry samples of the ‘Bain’ clone (18.06 ± 0.15 mg GAE/g DW, *p* < 0.05), and the highest total amount of flavonoids was detected in the ‘Drever’ and ‘Baiwfay’ cultivars (5.34 ± 0.026 mg RE/g DW and. 4.55 ± 0.30 mg RE/g DW) In order to clarify the variability in the content of individual phenolic compounds in the fruit samples of the studied American cranberry cultivars, we conducted the UPLC-ESI-MS/MS analysis. In the fruit samples of the studied cranberry cultivars, hyperoside, quercetin, and procyanidin A2 predominated among the identified phenolic compounds, while the amounts of other compounds were significantly lower. HCA and PCA revealed that fruit samples of ‘Woolman’, ‘Holliston’, ‘Pilgrim’, and ‘Searles’ cultivars had a different quantitative content of phenolic compounds from that in other cranberry cultivars. Meanwhile, fruit of ‘Baiwfay’, ‘Drever’, and ‘Bergman’ cultivars and the ‘Bain’ clone were similar in their phytochemical profiles. Fruit samples of the ‘Searles’ cultivar stood out among the others due to their exclusive phytochemical composition and strong antiradical (192.11 ± 0.99 µmol TE/g DW by ABTS assay) and reduction activity (38.68 ± 0.18 µmol TE/g DW by FRAP assay). Cranberry samples of the ‘Searles’ cultivar were found to have the highest total amount of the identified and quantitatively evaluated individual phenolic compounds (519.53 ± 25.12 mg/g DW). This cultivar could be selected as the desirable raw material for the preparation of cranberry fruit products.

## Figures and Tables

**Table 1:** Variability in the content of phenolic compounds in American cranberry fruit samples evaluated via the UPLC-ESI-MS/MS technique. The different letters indicate significant differences between the values.

Compound, mg/g	‘Bain’	‘Baiwfay’	‘Bergman’	‘Drever’	‘Holliston’	‘Pilgrim’	‘Searless’	‘Woolman’
Avicularin	5.26 ± 0.20 ^b^	2.99 ± 0.11 ^c^	7.17 ± 0.31 ^a^	5.72 ± 0.23 ^b^	2.85 ± 0.11 ^c^	2.77 ± 0.10 ^c^	6.01 ± 0.25 ^b^	5.52 ± 0.22 ^b^
Hyperoside	116.22 ± 5.30 ^b,c^	121.33 ± 4.36 ^b^	152.45 ± 6.96 ^a^	135.80 ± 6.60 ^a,b^	86.19 ± 3.89 ^c,d^	71.66 ± 3.16 ^d^	123.56 ± 5.56 ^a,b^	133.95 ± 6.37 ^a,b^
Isorhamnetin	25.68 ± 1.09 ^b,c^	28.03 ± 1.03 ^b,c^	23.92 ± 1.01 ^c^	30.43 ± 1.10 ^b^	9.73 ± 0.43 ^d^	10.27 ± 0.49 ^d^	39.08 ± 1.56 ^a^	11.42 ± 0.47 ^d^
Isorhamnetin-3-O-glucoside	10.96 ± 0.50 ^c,d^	14.81 ± 0.59 ^b^	15.98 ± 0.71 ^b^	20.99 ± 0.89 ^a^	8.85 ± 0.32 ^d^	7.68 ± 0.35 ^d^	14.61 ± 0.64 ^b^	12.71 ± 0.58 ^b,c^
Kaempferol	0.42 ± 0.02 ^a^	0.30 ± 0.02 ^b,c^	0.38 ± 0.02 ^a,b^	0.37 ± 0.02 ^a,b^	0.13 ± 0.01 ^e^	0.44 ± 0.02 ^a^	0.16 ± 0.01 ^d,e^	0.24 ± 0.01 ^c,d^
Quercetin	107.48 ± 4.79 ^b^	99.89 ± 3.98 ^b,c^	88.82 ± 3.89 ^b,c^	85.73 ± 3.85 ^c^	33.11 ± 1.45 ^d^	32.58 ± 1.50 ^d^	137.90 ± 6.03 ^a^	39.11 ± 1.88 ^d^
Quercitrin	49.95 ± 2.24 ^a^	56.67 ± 2.02 ^a^	47.25 ± 2.12 ^a^	47.05 ± 2.27 ^a^	31.10 ± 1.45 ^b^	23.71 ± 0.86 ^b^	48.70 ± 2.20 ^a^	51.26 ± 2.31 ^a^
Rutin	0.18 ± 0.01 ^c^	0.18 ± 0.01 ^c^	0.26 ± 0.01 ^c^	3.16 ± 0.14 ^a^	0.19 ± 0.01 ^c^	0.31 ± 0.01 ^c^	0.25 ± 0.01 ^c^	1.29 ± 0.05 ^b^
Luteolin-7-O-glucoside	0.11 ± 0.01 ^c,d^	0.08 ± 0.01 ^d^	0.25 ± 0.01 ^b^	0.15 ± 0.01 ^c,d^	0.85 ± 0.04 ^a^	0.18 ± 0.01 ^b,c^	0.11 ± 0.01 ^c,d^	0.15 ± 0.01 ^c,d^
(-)-Epicatechin	12.96 ± 0.58 ^a^	10.89 ± 0.47 ^a,b^	9.52 ± 0.42 ^b^	10.54 ± 0.49 ^a,b^	9.04 ± 0.38 ^b^	4.87 ± 0.21 ^c^	12.07 ± 0.57 ^a^	3.57 ± 0.15 ^c^
(+)-Catechin	3.60 ± 0.11 ^a,b^	2.60 ± 0.10 ^c,d^	3.10 ± 0.12 ^b,c^	4.18 ± 0.17 ^a^	3.55 ± 0.18 ^a,b^	1.11 ± 0.06 ^e^	4.28 ± 0.17 ^a^	2.18 ± 0.09 ^d^
Procyanidin A2	72.81 ± 3.21 ^c,d^	87.47 ± 3.85 ^b,c^	96.88 ± 3.98 ^a,b^	79.98 ± 3.52 ^b,c,d^	75.34 ± 2.80 ^c,d^	42.31 ± 1.98 ^e^	114.27 ± 5.32 ^a^	62.04 ± 2.79 ^d,e^
Procyanidin C1	0.96 ± 0.03 ^a,b^	0.95 ± 0.04 ^a,b^	0.90 ± 0.05 ^b,c^	0.80 ± 0.03 ^b,c^	0.77 ± 0.03 ^b,c^	0.47 ± 0.02 ^d^	1.16 ± 0.05 ^a^	0.70 ± 0.04 ^c^
Phloretin	0.14 ± 0.01 ^a,b^	0.13 ± 0.01 ^a,b,c^	0.11 ± 0.01 ^b,c^	0.12 ± 0.01 ^b,c^	0.13 ± 0.01 ^a,b,c^	0.08 ± 0.01 ^c^	0.15 ± 0.01 ^a,b^	0.18 ± 0.01 ^a^
Phloridzin	4.73 ± 0.17 ^d^	6.28 ± 0.28 ^b,c^	4.77 ± 0.19 ^d^	5.40 ± 0.18 ^c,d^	4.43 ± 0.18 ^d^	2.45 ± 0.11 ^e^	7.85 ± 0.36 ^a^	7.43 ± 0.33 ^a,b^
Gallic acid	0.51 ± 0.02 ^c,d^	0.40 ± 0.02 ^d^	1.14 ± 0.04 ^b^	0.64 ± 0.02 ^c^	1.36 ± 0.05 ^a^	0.37 ± 0.02 ^d^	0.52 ± 0.03 ^c,d^	0.97 ± 0.03 ^b^
Vanillic acid	0.89 ± 0.03 ^e^	1.95 ± 0.07 ^c^	2.04 ± 0.09 ^b,c^	1.45 ± 0.06 ^d^	1.15 ± 0.04 ^d,e^	0.75 ± 0.03 ^e^	2.46 ± 0.11 ^b^	3.18 ± 0.14 ^a^
Caffeic acid	0.44 ± 0.01 ^c^	0.21 ± 0.01 ^d,e^	0.80 ± 0.03 ^a^	0.26 ± 0.01 ^d^	0.09 ± 0.01 ^f^	0.15 ± 0.01 ^e,f^	0.25 ± 0.01 ^d^	0.54 ± 0.02 ^b^
Chlorogenic acid	1.52 ± 0.08 ^c,d^	2.69 ± 0.11 ^b^	4.64 ± 0.21 ^a^	2.57 ± 0.12 ^b^	2.15 ± 0.08 ^b,c^	1.10 ± 0.05 ^d^	4.26 ± 0.19 ^a^	2.42 ± 0.09 ^b^
Ferulic acid	0.71 ± 0.03 ^c^	0.68 ± 0.03 ^c^	1.12 ± 0.05 ^a^	1.10 ± 0.01 ^a^	0.70 ± 0.03 ^c^	0.93 ± 0.04 ^a,b^	0.77 ± 0.04 ^b,c^	0.72 ± 0.03 ^c^
Neochlorogenic acid	0.02 ± 0.001 ^c^	0.25 ± 0.01 ^a^	0.04 ± 0.01 ^c^	0.12 ± 0.01 ^b^	0.01 ± 0.001 ^c^	0.01 ± 0.001 ^c^	0.04 ± 0.002 ^c^	0.0004 ± 0.0001 ^c^
*p*-Coumaric acid	0.80 ± 0.02 ^e^	1.34 ± 0.05 ^b^	0.9 ± 0.03 ^d,e^	1.18 ± 0.04 ^b,c^	0.53 ± 0.03 ^f^	0.16 ± 0.01 ^g^	1.07 ± 0.04 ^c,d^	1.76 ± 0.06 ^a^
Total	416.35 ± 17.32 ^b^	440.13 ± 17.63 ^a,b,c^	462.42 ± 20.36 ^a,b^	437.73 ± 19.85 ^a,b,c^	272.23 ± 13.32 ^d^	204.36 ± 9.87 ^e^	519.53 ± 25.12 ^a^	341.32 ± 15.02 ^c^

**Table 2:** Mass spectrometry parameters for the analysis of phenolic compounds.

Compound	Parent Ion (*m/z*)	Daughter Ion (*m/z*)	Cone Voltage, V	Collision Energy, eV
*p*-Coumaric acid	163	93	28	22
Vanillic acid	167	152	26	12
Gallic acid	169	51	36	30
Caffeic acid	179	107	36	22
Ferulic acid	193	134	32	18
Phloretin	273	167	42	16
Kaempferol	285	185	50	25
(-)-Epicatechin	289	123	60	34
(+)-Catechin	289	123	60	34
Quercetin	301	151	48	20
Isorhamnetin	315	300	44	22
Chlorogenic acid	353	191	32	14
Neochlorogenic acid	353	191	32	14
Avicularin	433	301	50	20
Phloridzin	435	273	42	14
Luteolin-7-O-glucoside	447	285	66	26
Quercitrin	447	300	50	26
Hyperoside	463	300	50	26
Isorhamnetin-3-O-glucoside	477	314	60	28
Procyanidin A2	575	285	50	25
Rutin	609	300	70	38
Procyanidin C1	865,2	125	56	60
